# Beyond the Cascade Dams: Scale‐Dependent Effects on Zooplankton Stability in River Ecosystems

**DOI:** 10.1002/ece3.74019

**Published:** 2026-08-12

**Authors:** Xufei Jiang, Rixiu Zhou, Wenzhang Dai, Yipeng Yao, Yan Liu, Tianyi Sun, Jiachen Shen, Shuqing An, Lei Yan, Handan Zhang, Xin Leng, Zhi Ding

**Affiliations:** ^1^ Institute of Water Sciences Zhejiang University of Water Resources and Electric Power Hangzhou China; ^2^ School of Life Science and Institute of Wetland Ecology Nanjing University Nanjing China; ^3^ Qiantang River Basin Center of Zhejiang Province Hangzhou China

**Keywords:** cascade dams, community stability, different scale, zooplankton

## Abstract

Cascade dams are widely recognized as major drivers of environmental change in river ecosystems and are associated with alterations in biodiversity and community stability. However, the factors associated with community stability at different spatial scales remain unclear. This study compared zooplankton community synchrony and stability at local and regional scales and examined the factors associated with stability across these scales. We analyzed how variation in water quality and heavy metal factors is related to zooplankton diversity, synchrony, and stability using field sampling, community analysis, and structural equation modeling. Our results showed that cascade dams were associated with complex shifts in community stability and synchrony, and that these shifts varied notably between spatial scales. At the local scale, significant differences in community stability were observed between upstream, reservoir, and downstream sections of individual dams. Community stability was positively associated with diversity but showed no clear association with environmental variables. Improved water quality was associated with higher species richness and diversity, which in turn were associated with higher community stability and lower synchrony. At the regional scale, community stability generally increased from upstream to downstream, a pattern similar to that observed in undammed control rivers. Environmental variables were associated with both community structure and stability, with water quality showing positive associations and heavy metal factors showing negative associations. Overall, our findings highlight the importance of considering spatial scale when evaluating river ecosystem stability. Large‐scale cascade dam impacts may be associated with reduced stability through changes in water quality conditions.

## Graphical abstract

Cascade dams alter river zooplankton community stability, but how regulating factors act across local and regional spatial scales is poorly understood. Using field sampling and structural equation modeling, we revealed divergent scale‐dependent responses of zooplankton stability to dams—local stability depended on biodiversity, while regional stability was controlled by water quality and heavy metal pollution—demonstrating that spatial scale is essential for evaluating cascade dam ecological impacts.

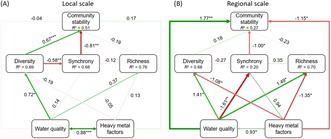

## 1 Introduction

Ecosystem stability is critical for sustaining ecological functions and services, a necessity that becomes even more critical as the impacts of global change intensify. Understanding the mechanisms that underpin ecosystem stability is therefore of urgent importance (Muraina et al. 2021). Studies have shown that ecosystem stability is influenced by a complex interplay of multiple factors, with biodiversity playing a central role. Typically, the greater number of species increases the likelihood of asynchrony in species' population fluctuations. This asynchrony can buffer the ecosystem against environmental variability (Hautier et al. 2014; Zhang, Loreau, et al. 2018). Additionally, species diversity can improve the stability of community attributes, such as total biomass and primary production (Thomas et al. 2020). Yet, this relationship is nuanced, influenced by variables such as species traits, functional redundancy, and habitat spatial configuration (Sasaki and Lauenroth 2011; Evans et al. 2022). Moreover, species synchrony—the alignment of population trends across species—plays a significant role in community stability (Muraina et al. 2021; Xuetao et al. 2022). High synchrony can reduce stability by magnifying the impact of adverse environmental changes on overall community dynamics (Donohue et al. 2016). Conversely, ecosystems where species populations fluctuate independently show greater resilience, a phenomenon known as compensatory dynamics, enabling adaptation and recovery from environmental stressors (Gonzalez and Loreau 2009; Hallett et al. 2017).

Recent studies have shown that the impact of various factors on community stability varies significantly across different scales (Zhang, He, et al. 2018). At the local scale, species interactions such as competition, predation, and mutualism significantly impact community stability. Conversely, at broader scales, aspects such as habitat heterogeneity, dispersal mechanisms, and environmental gradients become increasingly influential. Understanding how these factors interact at different scales is essential for a comprehensive grasp of ecosystem stability (Meng et al. 2023). However, the majority of current research has been centered on terrestrial ecosystems, with river ecosystems receiving comparatively less focus. This is particularly pronounced in river systems heavily influenced by human activities, such as the construction of cascade dams, which can modify species diversity, alter population dynamics, and impact ecosystem functions, ultimately affecting the long‐term stability of these communities (Bernhardt et al. 2022; Xie et al. 2022).

The construction of dams represents a significant human alteration to river ecosystems, fundamentally reshaping their ecological dynamics. Dams modify the physical properties of rivers, creating barriers to dispersion and altering levels of dissolved oxygen, nutrients, temperature, suspended sediment load, and flow regimes (Li et al. 2013; Erin et al. 2021; He et al. 2024). Similarly, cascade dams influence the watershed environment at different scales, affecting both community diversity and stability (Christian et al. 2017; Hamish et al. 2021). Nevertheless, the influence of environmental factors on community stability, particularly within river ecosystems, remains underexplored (Câmara et al. 2019; Thomas et al. 2020; Valencia et al. 2020; Zhang et al. 2022). Addressing this knowledge gap is crucial for advancing our understanding of ecosystem responses to global environmental changes, potentially guiding more effective conservation strategies.

Zooplankton, a crucial component of the aquatic food web, serve as valuable indicators for understanding broader ecological patterns and processes in river systems (Chang et al. 2024; Hu et al. 2024). Studies on zooplankton help illuminate the general principles of community stability in freshwater ecosystems and examine how stability is affected by a mix of biotic and abiotic factors. For instance, research has shown that temperature changes can alter zooplankton metabolic rates, which in turn affects their population dynamics (Fernández‐González and Marañón 2021). Likewise, the availability of nutrients influences the growth rate of phytoplankton, the primary food source for many zooplankton species (López‐Sandoval et al. 2021). Therefore, understanding the interaction between river community stability and various influencing factors at different scales necessitates a thorough examination of environmental characteristics.

The present study aims to compare the community synchrony and stability of zooplankton at local and regional scales, and to explore the factors influencing community stability across these different scales. This study proposes the following hypotheses: (1) The correlation between zooplankton community stability and synchrony differs between local and regional scales; (2) At the local scale, community diversity is the primary factor influencing community stability; (3) At the regional scale, community stability is primarily driven by environmental factors.

## 2 Methods

### 2.1 Research Location and Sample Collection

**FIGURE 1:**
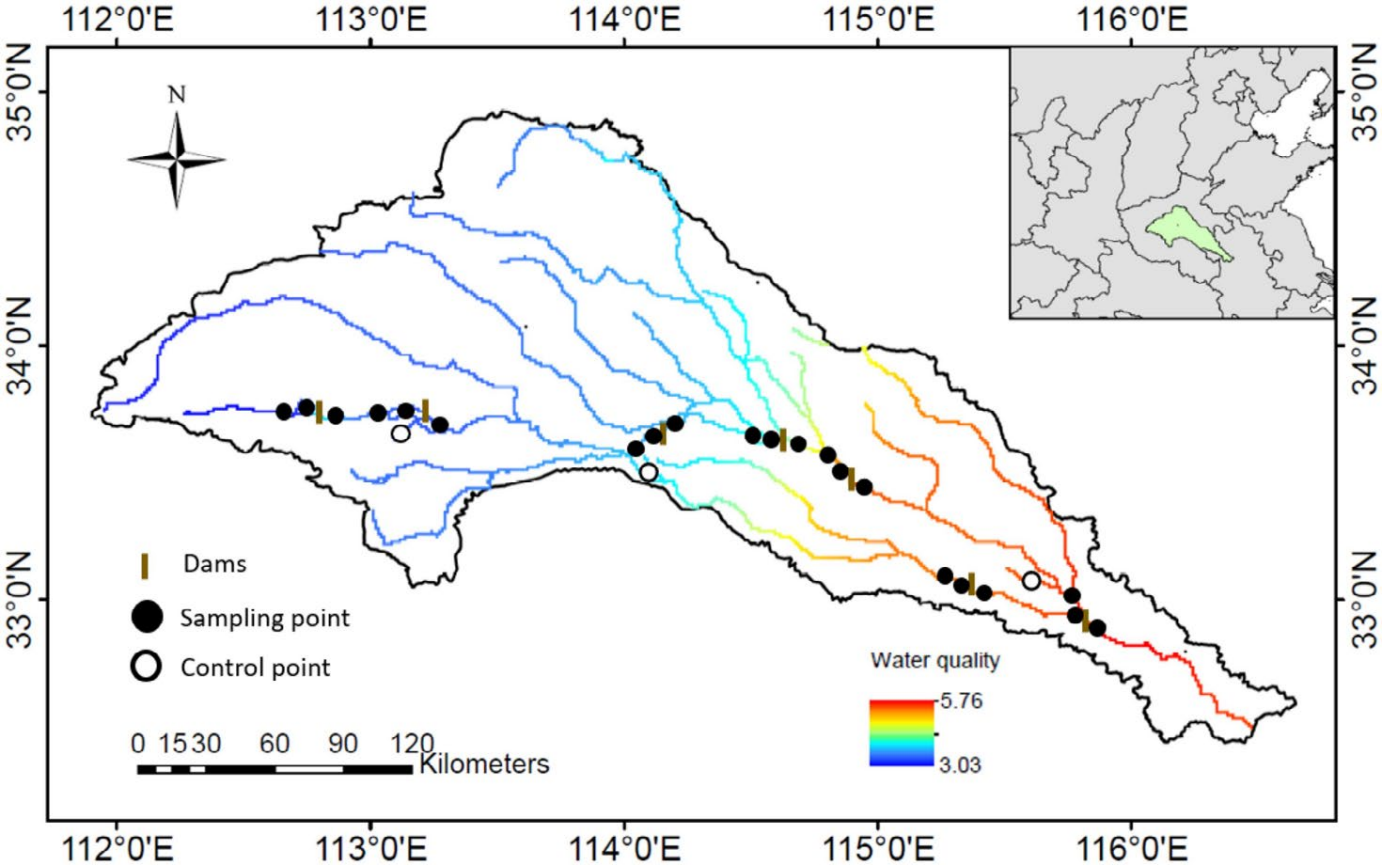
Sampling sites in the Shaying River Basin, with the river's color gradient indicating water quality.

In 2022, we conducted three field sampling campaigns (January, August, and November) across seven dams within the Shaying River basin. These included two upstream reservoirs (Zhaopingtai and Baiguishan), three midstream sluices or ship locks (Luohe, Zhoukou, Xiangcheng), and two downstream dams (Fuyang, Yangqiao). Three sample points were chosen at each dam, including upstream (more than 5 km from the dam within the stream's main channel), the reservoir center, and downstream (more than 5 km away from the reservoir). These 21 sites represented the local spatial scale for zooplankton research. The regional spatial scale was defined by the upstream, midstream, and downstream gradients of the entire basin (Figure 1). Additionally, a dam‐unregulated tributary was chosen as the control at different locations within the basin to compare zooplankton community differences between natural and dam‐impacted rivers.

Zooplankton sampling followed the standard protocol for freshwater zooplankton surveys in China (SC/T 9402‐2010). At each site, vertical hauls were conducted using a 64 μm mesh net (346 cm^2^ mouth area) from 0.5 m above the riverbed to the water surface and repeated five times. Vertical net tows were primarily used to obtain qualitative zooplankton samples representative of the entire water column, facilitating comprehensive species identification and improving the detection of large‐bodied and rare taxa. These qualitative samples complemented the quantitative samples obtained by filtering 25 L of water at each site. Protozoa, rotifers, and nauplii samples were concentrated to 30–50 mL and further settled for 24 h to obtain 10–20 mL subsamples. Cladocerans and copepods from net samples (~100 mL) were further concentrated when necessary depending on density. Samples were preserved using 40% formaldehyde (final concentration ~ 4%) for cladocerans and copepods, and Lugol's iodine solution (1.0%–1.5%) for protozoa and rotifers, with additional unfixed subsamples retained for live observation. Protozoa and rotifers' biomass were estimated using biovolume methods (specific gravity = 1.0), while cladocerans and copepods were quantified using body‐size‐based or direct measurement methods following SC/T 9402‐2010.

Counting procedures varied by taxon: protozoa were counted from 0.1 mL subsamples (two replicates), rotifers from 1 mL chambers (two replicates), and cladocerans/copepods from 5 mL chambers, with dilution applied when necessary. Large rotifers and nauplii were counted together with crustaceans depending on density. All samples were identified to the lowest possible taxonomic level (species or genus) under a microscope (100–400×), and abundance was standardized to individuals per liter (Jiang and Du 1979; Shen et al. 1979; Han and Shu 1995).

### 2.2 Water Quality and Heavy Metal Indicators Collection and Processing

In situ environmental parameters, including water temperature (WT), dissolved oxygen (DO), pH, electrical conductivity (EC), oxidation–reduction potential (ORP), total dissolved solids (TDS), and turbidity (NTU), were measured using HACH HQ40D portable water quality analyzer and HACH 2100q turbidimeter. Within 24 h of sampling, water samples were analyzed in the lab: chemical oxygen demand (COD), ammonium nitrogen (${\mathrm{NH}}_{4}^{+}-N$), total nitrogen (TN), and total phosphorus (TP) were determined via HACH DRB200 digester and HACH DR3900 spectrophotometer. For heavy metal analysis (Al, Fe, Pb, Hg, Cd, Cr, As, Zn), 1‐L water samples were tested using Thermo Fisher Scientific ICAP‐Q inductively coupled plasma mass spectrometry (ICP‐MS). The method detection limits for each parameter were as follows: COD 3 mg/L, ${\mathrm{NH}}_{4}^{+}-N$ 0.02 mg/L, TN 0.5 mg/L, and TP 0.02 mg/L. For heavy metals analyzed by ICP‐MS, detection limits were 0.002 μg/L. One method blank was included for every 5 samples, and data quality was verified with national standard samples (GNM‐M2610144‐2013). Finally, normalization was performed to eliminate magnitude differences among factors. We employed one‐way analysis of variance (ANOVA) to test for significant differences in environmental variables among different regions and visualized the results using Kriging interpolation in ArcGIS (ESRI, Redlands, CA, USA).

### 2.3 Community Structure Analysis and Stability Calculation

Zooplankton assemblages were classified into four major taxonomic groups: Protozoa, Rotifera, Copepoda, and Cladocera. To evaluate spatial variation in community structure, we quantified abundance, species richness, and biomass for each group across sampling sites. Differences in community structure among spatial units were assessed using one‐way analysis of variance (ANOVA) and Kruskal–Wallis tests, and visualized in R. Species richness and Shannon diversity were calculated based on pooled species abundance data from three seasonal sampling campaigns conducted in January, August, and November 2022.

Community stability was quantified as temporal stability of total zooplankton abundance in R with the “codyn” package (Hallett et al. 2016), calculated as the ratio of the temporal mean to its standard deviation (mean/SD) across three seasonal sampling campaigns (January, August, and November 2022). Variance ratio and species synchrony were additionally used as complementary metrics to describe compensatory population dynamics (Gouhier and Guichard 2014; Hallett et al. 2014, 2016).

The variance ratio compares the overall community variance (C) with the sum of individual species (*xi*) variances:

$$\mathrm{VR}=\frac{\mathrm{Var}\left(C\right)}{\sum_{i}^{N}\mathrm{Var}\left(\mathrm{xi}\right)}$$

and

$$\mathrm{Var}\left(C\right)=\sum_{i=1}^{N}\mathrm{Var}\left(\mathrm{xi}\right)+2\;\left(\sum_{i=1}^{N-1}\sum_{j=i+1}^{N}\mathrm{Cov}\left(\mathrm{xi},\mathrm{xj}\right)\right)$$

Loreau and de Mazancourt proposed a species synchrony index (Loreau and de Mazancourt 2008), comparing the variance of the sum of species abundances with the sum of individual species variances:

$$\text{Synchrony}=\frac{\sigma {\left(x_{T}\right)}^{2}}{{\left(\sum_{i}\sigma_{\mathrm{xi}}\right)}^{2}}$$

and

$$x_{T}\left(t\right)=\sum_{i=1}^{N}\mathrm{xi}\left(t\right)$$

This synchrony calculation is standardized between 0 (completely asynchronous) and 1 (completely synchronous), and the advantage of this index is that it can be applied to communities with different species richness.

### 2.4 Statistical Analysis

One‐way analysis of variance (ANOVA) and Kruskal–Wallis tests were applied to compare differences in zooplankton community stability and synchrony across scales. ArcGIS (ESRI, Redlands, CA, USA) Kriging interpolation was used to visualize the spatial pattern of zooplankton community stability across the basin. Linear regression was employed to explore relationships between community stability, variance ratio, and synchrony. Univariate regression analysis (via R “vegan” and “ggplot2” packages) examined how diversity/richness affected stability. Correlation analysis and Mantel tests (via R “Hmisc” and “ggcor” packages) evaluated impacts of water quality and heavy metals.

One‐way analysis of variance (ANOVA) and Kruskal–Wallis tests were applied to compare differences in zooplankton community stability and synchrony across spatial scales. ArcGIS (ESRI, Redlands, CA, USA) Kriging interpolation was used to visualize the spatial distribution of community stability across the basin. Linear regression was employed to examine relationships between community stability, variance ratio, and synchrony. In addition, univariate regression analyses (using the R packages “vegan” and “ggplot2”) were conducted to explore the relationships between diversity/richness and stability. Correlation analyses and Mantel tests (using the R packages “Hmisc” and “ggcor”) were used to evaluate associations between environmental variables (water quality and heavy metals) and zooplankton community metrics.

To reduce multicollinearity and improve interpretability, environmental variables were grouped into two composite indices: water quality (WQ) and heavy metals (HM). The WQ index included WT, ORP, NTU, TP, DO, TN, pH, TDS, and conductivity, while the HM index included Fe, Pb, As, Cd, Zn, and Cr. These variables were selected based on ecological relevance and prior correlation screening to retain those showing relatively strong associations with zooplankton community metrics. All variables were standardized prior to aggregation, and composite indices were calculated as the sum of standardized values.

Structural equation modeling (SEM) was conducted using the R package lavaan to evaluate potential direct and indirect associations among environmental gradients (WQ and HM), community diversity (richness and Shannon diversity), synchrony, and stability at local and regional scales. Model selection was based on goodness‐of‐fit indices (*χ* ^2^, CFI, RMSEA, SRMR) together with Akaike Information Criterion (AIC), and the model with the best overall performance was selected.

## 3 Results

### 3.1 Environmental Characteristics and Zooplankton Community Structure

Environmental variables exhibited significant spatial variation among dam sections and across the watershed. EC, TDS, TN, TP, NH_4_^+^‐N and several heavy metals (e.g., As and Cr) differed significantly among sampling locations. Water quality and heavy metal indices generally increased from upstream to downstream reaches of dam‐regulated rivers. Detailed environmental characteristics and statistical comparisons are provided in Figures S1 and S2 and Table S1; these data were reported previously (Jiang et al. 2023).

**FIGURE 2:**
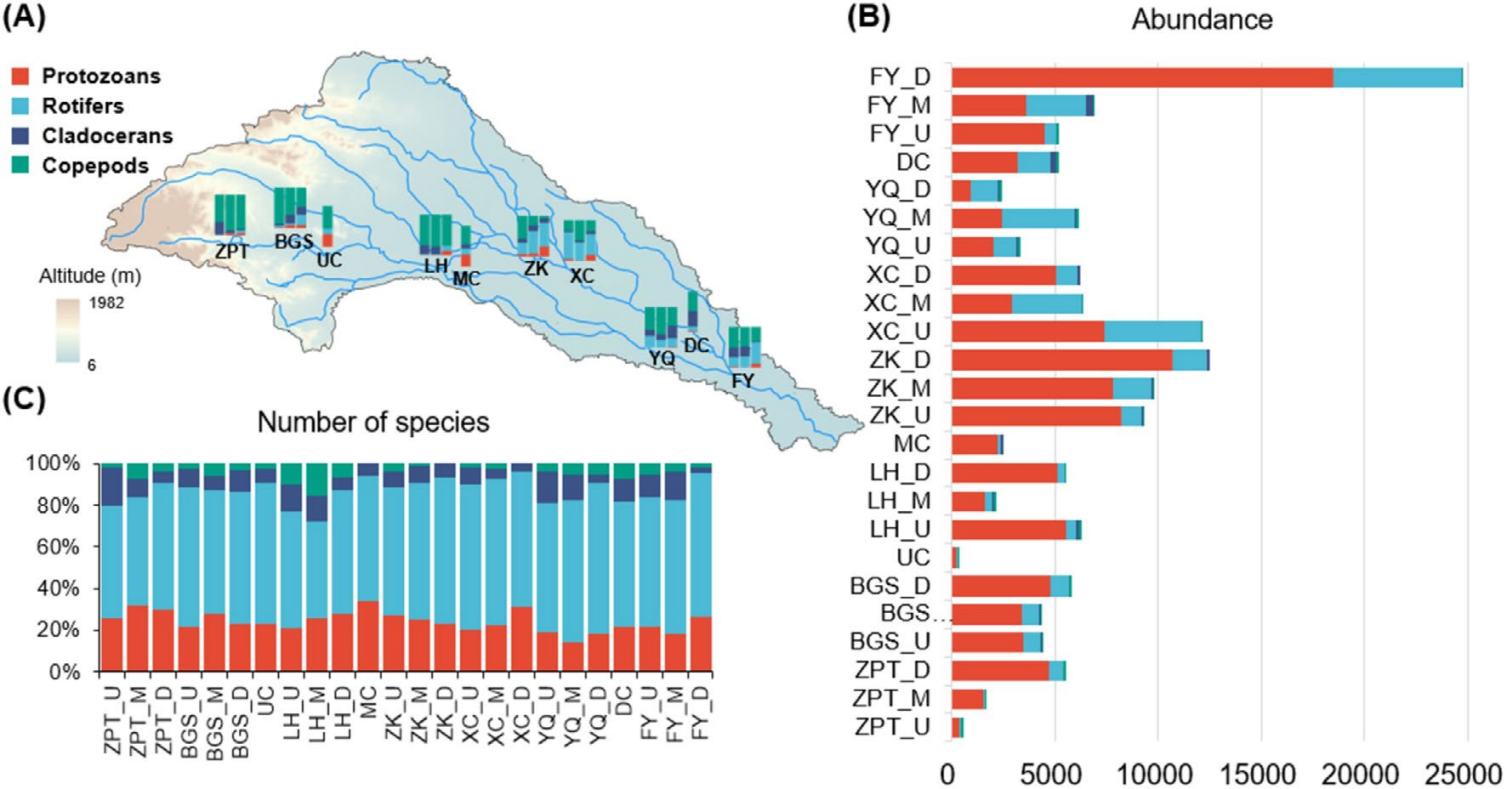
Zooplankton community structure at the local scale. (A) Proportional biomass of zooplankton across Seven dams and three control groups. This includes protozoans, rotifers, copepods, and cladocerans. (B) Community abundance across different sampling sites. (C) Differences in the number of zooplankton species. D represents downstream of the dam, M indicates midstream of the dam, U signifies upstream of the dam, DC denotes the downstream control group, MC stands for the midstream control group, and UC represents the upstream control group.

A total of 127 zooplankton species were identified across the Shaying River Basin, including 37 protozoan species, 67 rotifer species, 17 cladoceran species, and 6 copepod species. At the local scale, zooplankton biomass varied considerably among sampling locations associated with dams. The highest biomass was recorded downstream of the Fuyang Dam, whereas the lowest biomass occurred upstream of the Zhaopingtai Reservoir. Copepods contributed most of the biomass at Zhaopingtai Reservoir and Luohe Dam, whereas rotifers dominated biomass at Zhoukou and Xiangcheng dams (Table S2). Marked differences in biomass composition were observed between dam‐regulated sites and corresponding control reaches. The relative biomass of protozoa in the upstream and midstream control groups was substantially higher than that observed at Baiguishan, Zhaopingtai, Luohe, Zhoukou, and Xiangcheng dams, with the exception of the downstream site of Zhoukou Dam. In contrast, the biomass composition of the downstream control group was more similar to that observed at downstream Yangqiao Dam and the upstream and reservoir‐center sections of Fuyang Dam (Figure 2A). Zooplankton abundance also differed substantially among local‐scale sites. The highest abundance was recorded downstream of Fuyang Dam, followed by downstream Zhoukou Dam, with protozoa accounting for the majority of individuals. The lowest abundance was observed upstream of Zhaopingtai Reservoir. In addition, zooplankton abundance in the upstream and midstream control groups was generally lower than that in dam‐regulated reaches. Species composition analysis revealed that rotifers represented the most species‐rich group throughout the basin, followed by protozoa (Figure 2C).

**FIGURE 3:**
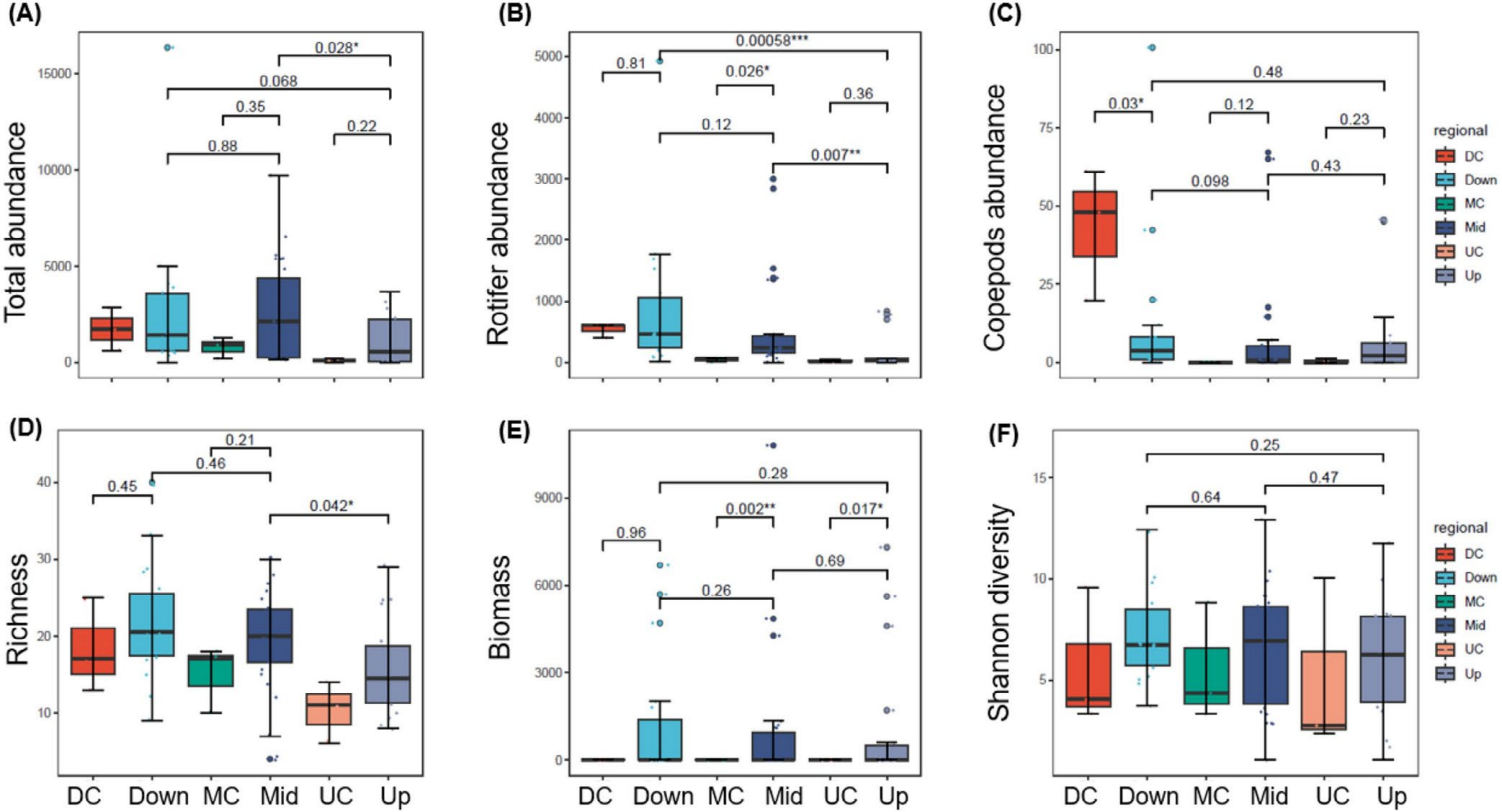
Zooplankton communities at the regional scale for (A) total abundance, (B) rotifer abundance, (C) copepod abundance, (D) richness, (E) biomass, and (F) Shannon diversity.

At the regional scale, significant differences were detected in both species richness and zooplankton abundance among river sections (*p* = 0.04 and *p* = 0.03, respectively). In particular, richness and abundance in the middle reaches were significantly higher than those in the upstream reaches. Furthermore, zooplankton richness was significantly greater in dam‐regulated rivers than in corresponding control reaches (Figure 3A,D). Rotifers were identified as the primary contributors to regional differences in community structure. Their abundance increased progressively from upstream to downstream reaches, with highly significant differences between the upstream region and both the middle and downstream regions (Figure 3B). Comparisons among river sections showed that copepod abundance was significantly higher in dam‐regulated reaches than in control reaches in the upstream and middle reaches. However, in downstream reaches, copepod abundance was significantly greater in control rivers than in dam‐regulated rivers (Figure 3C). Both zooplankton richness and biomass exhibited increasing trends from upstream to downstream sections of the basin and were generally higher in dam‐regulated rivers than in control reaches (Figure 3D,E).

### 3.2 Community Synchrony and Stability at Different Scales

**FIGURE 4:**
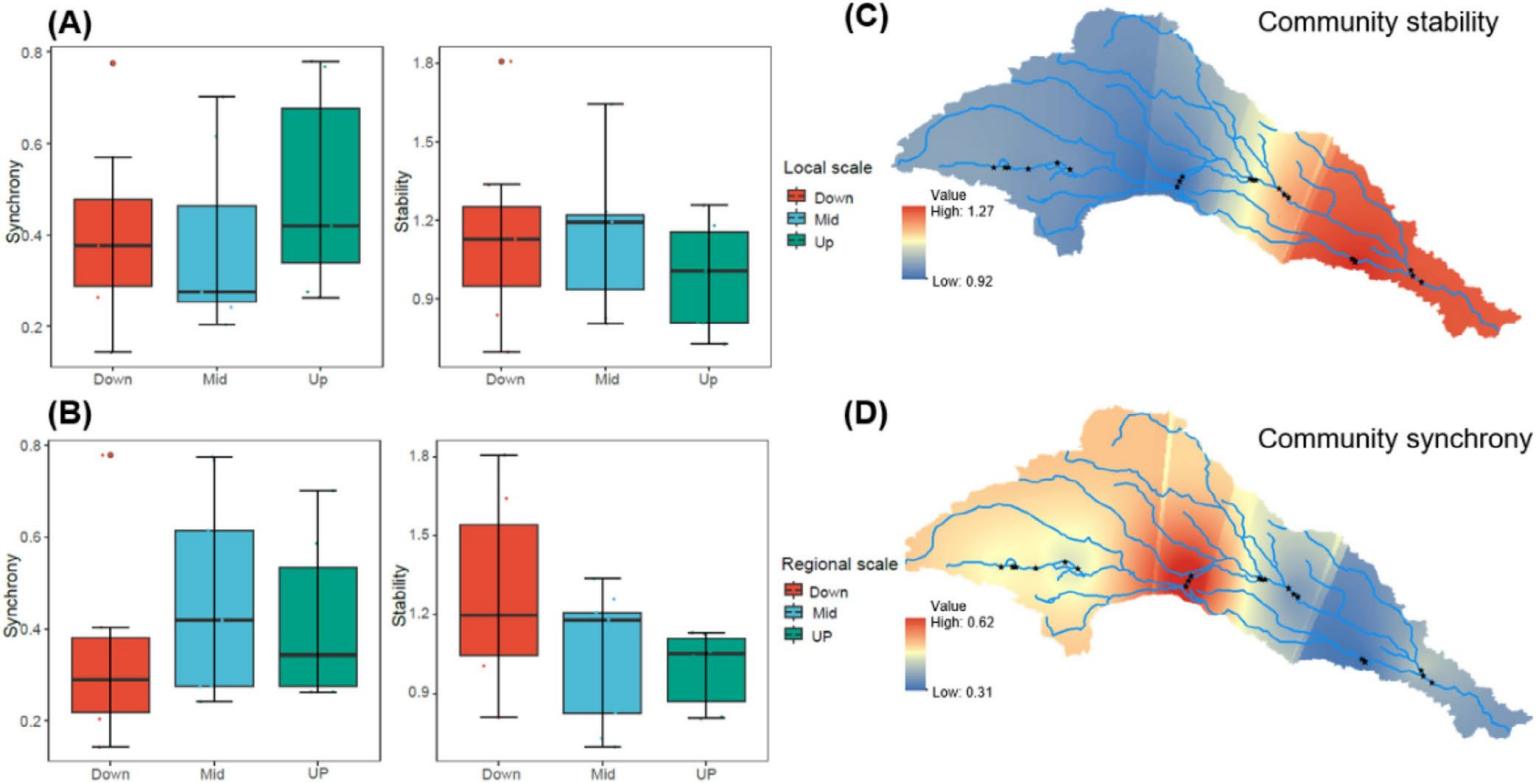
(A) Boxplots of synchrony and stability of upstream, midstream, and downstream communities of dams at local scale. (B) Boxplots of synchrony and stability of communities in the up, mid, and downstream of the watershed at the regional scale. (C) Community stability across the entire watershed depicted using Kriging interpolation. (D) Variations in community synchrony within the watershed displayed using Kriging interpolation.

At the local scale, community synchrony was higher in the upstream of the dam than in the downstream and midstream; in contrast, average community stability was highest in the midstream, followed by the downstream and upstream (Figure 4A). The highest community stability was observed downstream of the Yangqiao Dam and midstream of the Fuyang Dam, while the upstream and downstream areas of the Luohe Dam displayed lower stability levels. This trend inversely mirrored community synchrony patterns (Figure 4). Notably, significant variations in community stability were observed even within small distances of about 5 km around a dam, particularly in the midstream and upstream areas of the Fuyang Dam and throughout the Yangqiao Dam.

At the regional scale, the midstream of the basin had the highest synchrony, but community stability was highest in the downstream and lowest in the midstream (Figure 4B). Meanwhile, Kriging interpolation maps showed a clear trend of increasing community stability from upstream to downstream within the basin, paralleling the pattern observed in rivers without dams (Figure 4C). In contrast, while community synchrony decreased progressively in control rivers, dam‐affected regions exhibited the highest synchrony in midstream, decreasing toward the downstream areas (Figure 4D).

**FIGURE 5:**
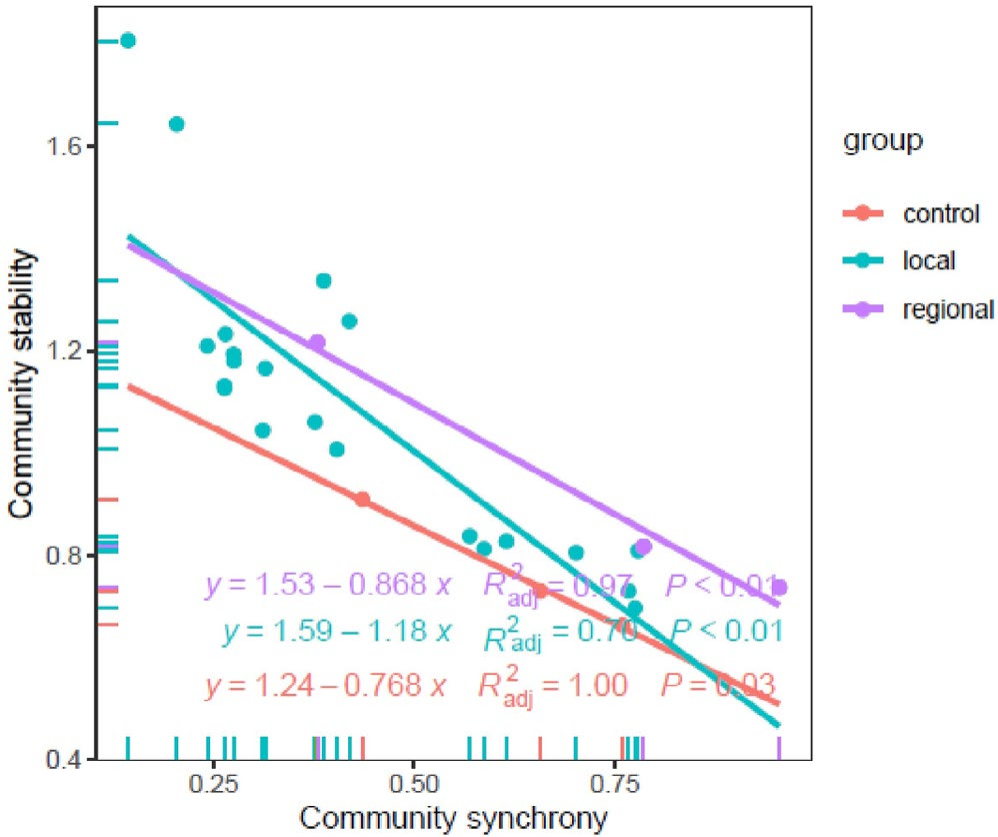
Relationships between community stability and synchrony across different scales.

**FIGURE 6:**
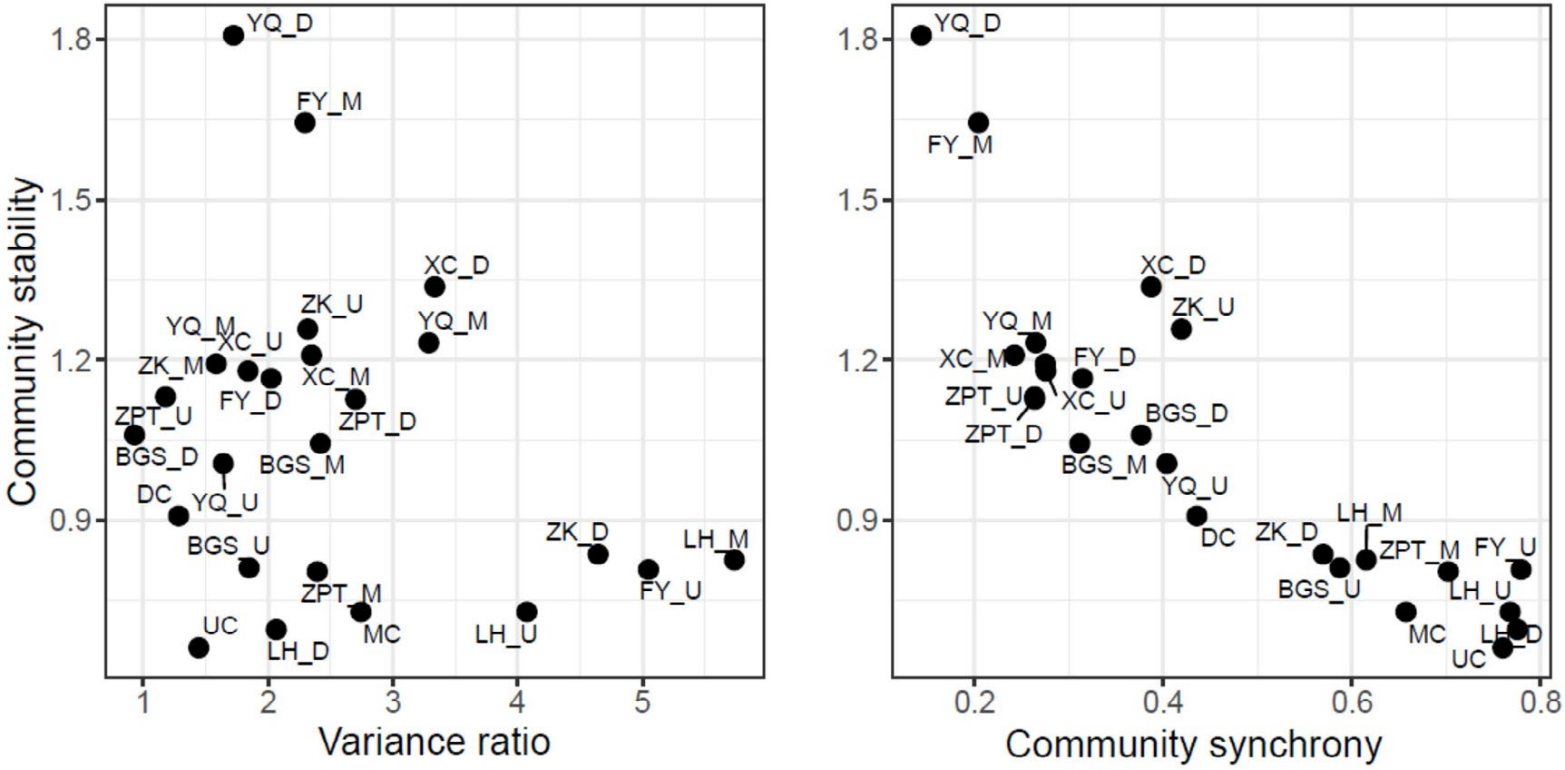
Relationships between community stability, variance ratio, and synchrony at the local scale.

Statistical analyses revealed a significant negative correlation between community stability and synchrony, with the correlation becoming stronger at local scale than regional scale (Figure 5). At the local scale, there was no significant difference between community stability and the variance ratio, with community stability only showing a significant negative correlation with community synchrony (Figure 6).

### 3.3 The Relationship Between Diversity, Environment and Stability at Different Scales

**FIGURE 7:**
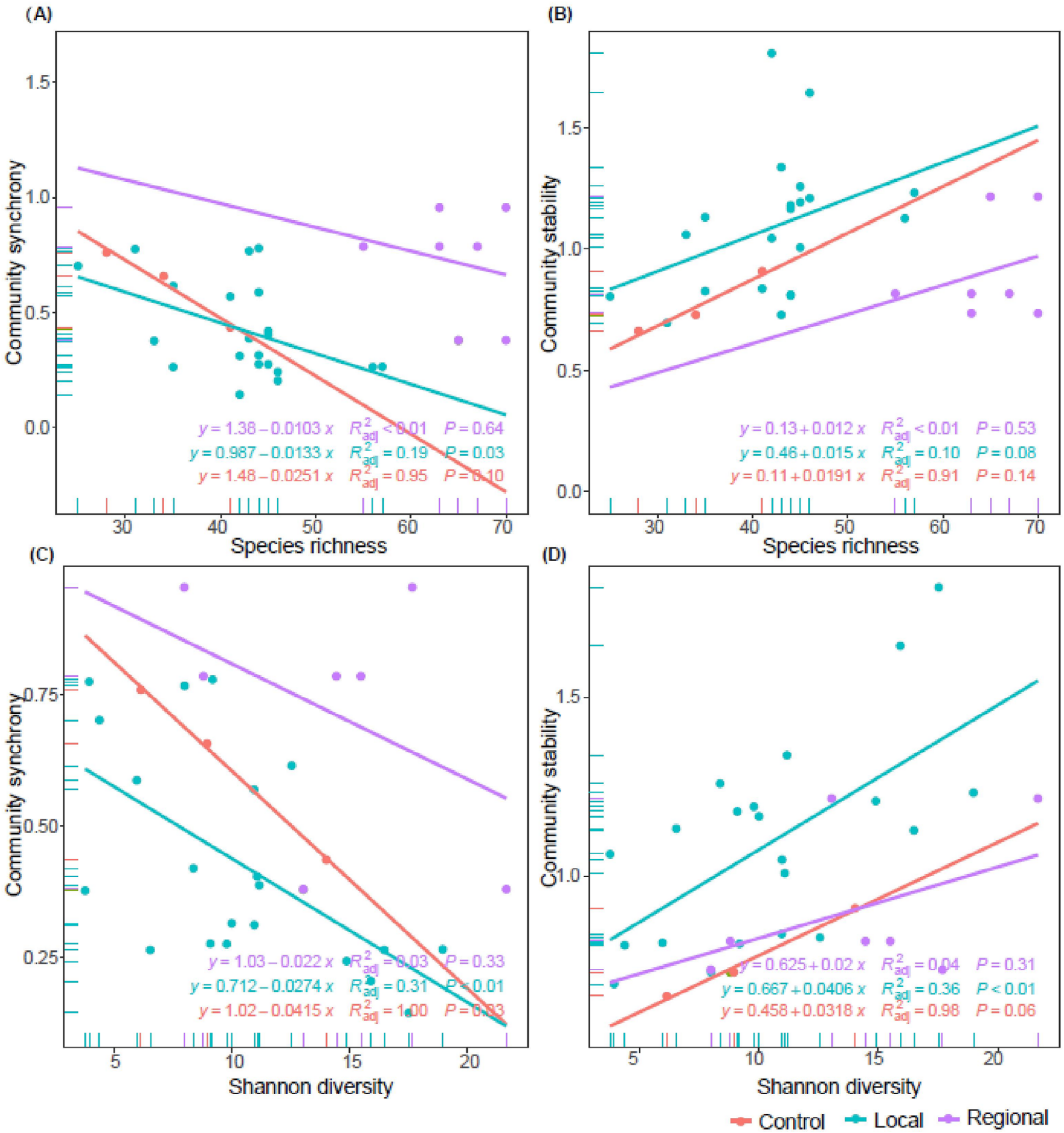
Relationships at different scales: (A) between species richness and community synchrony, (B) between richness and community stability, (C) between community diversity and synchrony, and (D) between community diversity and stability.

Our analyses revealed distinct scale‐dependent patterns in how biodiversity metrics and environmental factors influenced community stability (Figure 7). At the local scale, both species richness (*p* = 0.03) and Shannon diversity (*p* < 0.01) exhibited significant negative correlations with community synchrony (Figure 7A,C), while showing strong positive associations with stability (*p* < 0.01; Figure 7B,D). However, no significant environmental influences on stability were detected at this scale (Figure 7).

**FIGURE 8:**
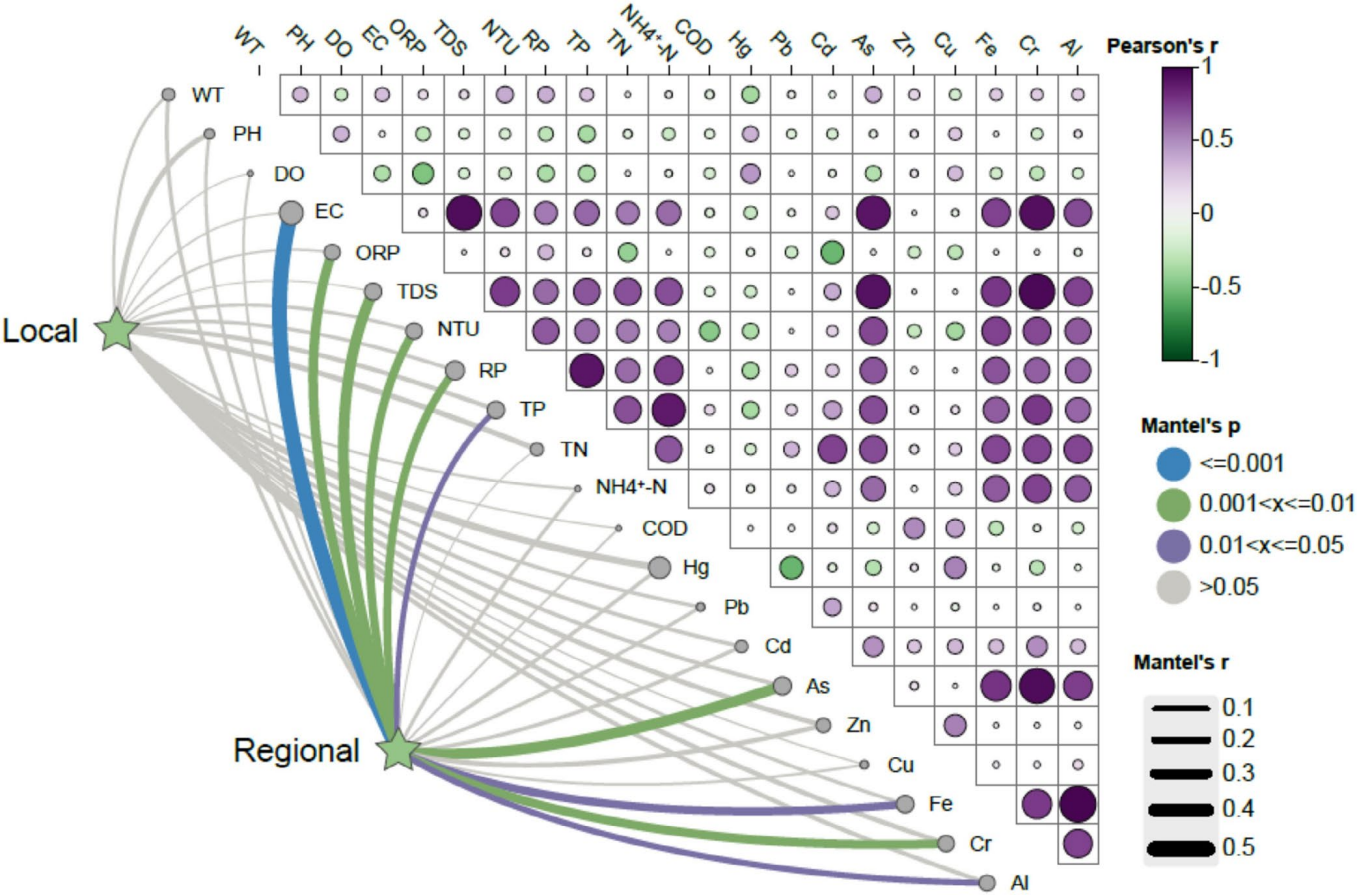
Impact of water quality and heavy metal indicators on community stability between different scales. Line thickness indicates the strength of correlation between stability and environmental factors. Each optimal model is represented by lines in blue (*p* < 0.001), green (*p* < 0.01), and purple (*p* < 0.05), with correlations among environmental factors shown on the right.

In contrast, regional scale patterns demonstrated fundamentally different drivers. While biodiversity metrics showed no direct correlation with stability, multiple environmental factors emerged as significant predictors. Electrical conductivity (EC: *r* = 0.54, *p* = 0.001), oxidation–reduction potential (ORP: *r* = 0.32, *p* = 0.004), and total dissolved solids (TDS: *r* = 0.37, *p* = 0.002) showed particularly strong positive associations with stability. Heavy metal factors displayed complex relationships, with arsenic (As: *r* = 0.37, *p* = 0.003) and chromium (Cr: *r* = 0.29, *p* = 0.008) showing positive correlations, while others like aluminum (Al: *r* = 0.18, *p* = 0.04) exhibited weaker associations (Figure 8).

### 3.4 Scale‐Dependent Structural Equation Modeling of Community Stability and Its Associated Factors

The structural equation models showed acceptable goodness‐of‐fit at both local and regional scales. At the local scale, the model fit was adequate (*χ* ^2^ = 11.642, df = 1, *p* = 0.001; CFI = 0.863; SRMR = 0.045; RMSEA = 0.071). At the regional scale, model performance was also satisfactory (*χ* ^2^ = 6.359, df = 1, *p* = 0.012; CFI = 0.922; SRMR = 0.037; RMSEA = 0.087; GFI = 1.000).

**FIGURE 9:**
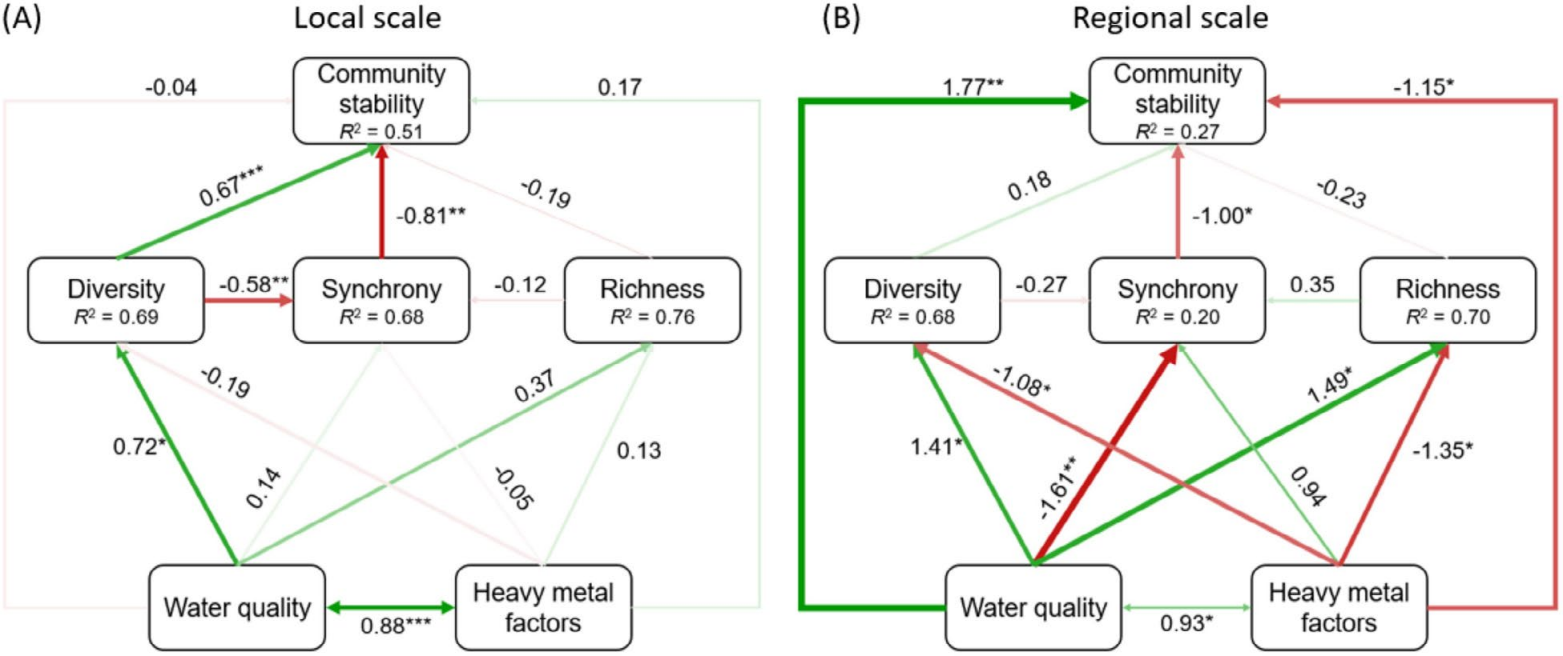
Structural equation models describing scale‐dependent associations among environmental variables, community structure, synchrony, and stability of zooplankton communities at (A) local and (B) regional scales. Standardized path coefficients are shown adjacent to arrows, representing the strength and direction of relationships among variables. Red and green arrows indicate negative and positive associations, respectively. Arrow width is proportional to the magnitude of standardized path coefficients. Significance levels are indicated as follows: *p < 0.05, **p < 0.01, ***p < 0.001.

SEM results revealed scale‐dependent differences in the associations among environmental variables, community structure, and stability‐related metrics. At the local scale, water quality was strongly associated with community diversity. Higher diversity was positively associated with community stability and negatively associated with community synchrony. In addition, community synchrony was negatively associated with stability, indicating consistent statistical associations among diversity, synchrony, and stability at this scale. At the regional scale, environmental variables showed broader associations with both community structure and stability. Water quality was positively associated with diversity and richness, whereas heavy metal variables were negatively associated with these metrics. Water quality was positively associated with community stability and negatively associated with synchrony, while heavy metals showed the opposite pattern. At this scale, richness and diversity did not show clear direct associations with community stability, indicating scale‐dependent differences in the statistical relationships among environmental conditions, community structure, and stability‐related metrics (Figure 9).

## 4 Discussion

### 4.1 Scale‐Dependent Differences in Zooplankton Community Structure

The effects of cascade dams on zooplankton community structure are inherently complex, and our results highlight the importance of examining community responses across multiple spatial scales. At the local scale, dam regulation was associated with pronounced differences in zooplankton biomass and abundance among upstream, reservoir‐center, and downstream sections. Zooplankton biomass generally peaked within reservoir sections, likely owing to reduced flow velocity and increased water residence time, which create relatively stable conditions favorable for plankton growth and accumulation (Battauz et al. 2017). In contrast, zooplankton abundance and community composition in upstream sections more closely resembled those of unregulated control reaches, consistent with previous studies indicating that upstream areas often retain hydrological characteristics closer to natural river conditions (Baubekova et al. 2023).

Despite these similarities, protozoan biomass accounted for a significantly larger proportion of total biomass in control reaches than in reservoir‐center and downstream sections. As protozoa are particularly sensitive to hydrological and environmental disturbances (Guermazi et al. 2023), their greater contribution in control sites may reflect habitat conditions that remain closer to the natural state. Furthermore, clear differences between dam‐regulated and control reaches suggest that dam operation alters not only local habitat conditions but also the taxonomic composition of zooplankton communities. Such differences among zooplankton groups may arise from variation in environmental sensitivity, life‐history traits, and responses to altered hydrological regimes. In particular, rotifers and protozoa are characterized by rapid population turnover and may respond more quickly to environmental fluctuations associated with dam regulation, potentially contributing to the elevated species richness observed within reservoir sections.

At the regional scale, variation in community structure was primarily driven by differences in rotifer and copepod abundance, as well as overall species richness. Reservoirs in the upper watershed exhibited relatively simple communities dominated by copepods, a pattern that may be related to longer water residence times and comparatively better water quality conditions that favor copepod development (Guo et al. 2016). In contrast, rotifers contributed a larger proportion of community biomass in the more urbanized middle and lower reaches, where dense dam construction and intensified anthropogenic activities may create highly variable environmental conditions. Their short generation times and high reproductive rates likely enable them to respond rapidly to such disturbances (Bai et al. 2021). Across the watershed, zooplankton abundance and biomass generally increased from upstream to downstream reaches, indicating cumulative ecological responses along the river continuum. Cascade dams modify hydrological conditions directly and may further influence zooplankton communities indirectly through changes in water quality, sediment dynamics, and habitat connectivity (Li et al. 2019). The lowest abundance and richness were observed in upstream reservoirs, where prolonged water retention and increased water depth may intensify hydraulic loading effects and reduce plankton diversity (Eva et al. 2005; Meiling et al. 2020). Notably, differences between dam‐regulated and control reaches were more pronounced at the regional scale than at the local scale, suggesting that the ecological consequences of cascade dams emerge not only through localized habitat modification but also through cumulative effects operating across the broader river network (Zhang et al. 2010; Gilmar et al. 2011).

These findings emphasize that assessments of ecological responses to cascade dams should incorporate regional‐scale perspectives in addition to local‐scale analyses. Scale‐dependent differences in community composition, abundance, and diversity may further influence patterns of species synchrony and community stability, providing an important ecological foundation for understanding how cascade dams affect the stability of riverine zooplankton communities.

### 4.2 Scales Influence Zooplankton Synchrony and Stability

Our research findings revealed that community synchrony and stability in river ecosystems vary significantly between different scales. At the local scale, we observed significant differences in community stability between upstream and downstream areas of the dam, especially at the Yangqiao and Fuyang Dams. Meanwhile, the reservoir center appeared to be the area of lowest community synchrony and highest stability. This might occur because of the relatively stable hydrological environment in the dam impoundment area, where the community is less disturbed. In contrast, the upstream and downstream of dams are exposed to sharp water level rise and flood discharge scour, respectively (Liu et al. 2009; Cui et al. 2022). These diverse hydrological conditions and human interventions highlight the effects of dams on community stability at small scales (Yang et al. 2019; Boulange et al. 2021). At the regional scale, community stability tended to increase from upstream to downstream, similar to patterns observed in undammed rivers, indicating that the effects of a single dam on community stability may be mitigated at larger scales (Wang et al. 2019; Hasan 2023). For instance, a large reservoir might mitigate the flooding risks posed by all upstream reservoirs, thereby reducing overall system instability (Yang et al. 2017; Wang et al. 2023). In contrast to the gradual decline in synchrony from upstream to downstream observed in the control group, the highest synchrony was observed in the midstream. This suggests that cascade dams exert a unique influence on community synchrony through alterations to flow regimes and environmental conditions, particularly in areas where dams and urban development were concentrated (Shen et al. 2022).

Moreover, our study revealed a significant negative correlation between community stability and synchrony at both local and regional scales, in line with prior research on forests or grasslands (Vazquez et al. 2009; Valencia et al. 2020). This demonstrates that asynchrony between populations in river ecosystems similarly enhances overall community stability. Furthermore, these patterns suggest that at the local scale, the correlation between synchrony and stability was stronger, while the correlation between these two at the regional scale was closer to that of undammed rivers. This suggests that in dammed rivers, akin to certain terrestrial ecosystems, community stability at smaller scales is predominantly driven by synchrony (Wilcox et al. 2017; Zhang, He, et al. 2018; Valencia et al. 2020). However, cascade dams introduce more complex factors influencing community stability at regional scales.

### 4.3 Diversity and Environmental Variables Related to Stability at Different Scales

Species richness and diversity are widely recognized as key drivers of ecological stability (de Mazancourt et al. 2013; Hallett et al. 2014; Zhang et al. 2016). In undammed rivers, the relationship between community stability and increasing species diversity was typically more pronounced than in rivers impacted by dams. This could be attributed to the stronger natural resilience and adaptability of communities in undammed rivers, which allow them to maintain relatively stable structures despite natural fluctuations such as water level changes and floods (Tonkin et al. 2017). In contrast, cascade dams significantly altered environmental conditions—including flow regimes, water quality, and habitat structures—potentially diminishing the positive effects of biodiversity on ecosystem stability (Yang et al. 2017; Sabater et al. 2018). Although the direct correlation between community stability and species diversity in undammed rivers was not significant, the observed steeper slope indicates a potential role for species diversity in maintaining ecosystem functions, likely due to enhanced complementary actions and functional redundancy (Bluethgen et al. 2016).

While previous studies emphasized biodiversity's universal stabilizing role (Thompson et al. 2015; Bluethgen et al. 2016; Pennekamp et al. 2018; Evans et al. 2022), our work demonstrates this principle's spatial boundedness in dam‐regulated rivers. Our research suggested that species richness and diversity were more strongly correlated with stability at local scale, while the direct correlation was weak at regional scale. Notably, many environmental variables were strongly correlated with community stability at the regional scale. The health of zooplankton communities was contingent upon the water quality and metal ion factors (Baliarsingh et al. 2010; Alprol et al. 2022). For instance, phosphorus content showed a significant correlation with community stability, was associated with higher productivity indicators and zooplankton proliferation (Jochimsen et al. 2013; Song et al. 2021). Mercury and other heavy metals also play a significant role, particularly due to their accumulation in the water from dam storage (He et al. 2022). At the regional scale, stability was strongly correlated with several environmental variables such as electrical conductivity, oxidation–reduction potential, and turbidity. These factors were impacted by human activities, such as the discharge of sewage and upstream floodwaters, which exacerbated changes in water quality (Sabater et al. 2018; Wang et al. 2020; Bao et al. 2021; Lv et al. 2021). Notably, after dam discharges in the upstream to downstream of the basin, increasing of sediment exposure times may lead to increased concentrations of heavy metals such as As, Fe, Cd, Ni, and Zn, altering water chemistry and affecting community stability (Rios‐Arana et al. 2004; Hahn et al. 2018). These heavy metals often co‐vary with anthropogenic nutrient inputs in the basin; the associated increase in phytoplankton productivity may indirectly support higher zooplankton community stability, leading to an apparent positive correlation between metal concentrations and stability. Thus, the presence of cascade dams intensifies the accumulation of heavy metals, significantly impacting community stability at the regional scale.

### 4.4 Scale‐Dependent Associations Between Environmental Factors and Community Stability

Using structural equation modeling (SEM), scale‐dependent differences were identified in the associations among environmental factors, community structure, and stability‐related metrics. At the local scale, community stability was more closely associated with community diversity and synchrony. Hydrological regulation by dams may reduce habitat heterogeneity, which is associated with variation in community diversity (Wu et al. 2004; Baird and Hogan 2023). Stable species composition is often considered an important component of maintaining community stability in riverine ecosystems (Sasaki and Lauenroth 2011). Accordingly, higher species diversity may be associated with more stable community dynamics at the local scale. In addition, diversity was positively associated with water quality, consistent with previous studies (Bera 2021; Guermazi et al. 2023; Mohammed et al. 2023). This suggests that environmental conditions may indirectly relate to stability at local scales, where nutrient accumulation in dammed systems may be associated with increased diversity, which in turn is linked to reduced synchrony and higher community stability.

In contrast to many terrestrial ecosystems (Zhang, He, et al. 2018; Zhang et al. 2019; Meng et al. 2023), at the regional scale, community stability showed stronger associations with environmental variables than with species richness or diversity, as demonstrated in previous zooplankton studies (Zhang et al. 2022). Water quality was positively associated with species richness, diversity, and community stability, whereas heavy metal variables were negatively associated with these metrics. In addition, environmental variables may indirectly relate to stability through their associations with community synchrony. Nutrient inputs may be linked to species turnover and community dynamics (Jochimsen et al. 2013), whereas elevated heavy metal concentrations may be associated with reduced community performance and altered community structure. Overall, these results highlight scale‐dependent differences in the statistical associations among environmental conditions, biodiversity, and community stability in river ecosystems.

## 5 Conclusions

This study highlights scale‐dependent differences in the associations between environmental conditions, biodiversity, and community stability. At the local scale, dam‐related environmental changes were associated with variation in community diversity, which was in turn associated with community stability. At the regional scale, community stability showed stronger associations with environmental variables than with biodiversity metrics.

These findings emphasize the importance of considering spatial scale when evaluating river ecosystem stability. For river management, both biodiversity conservation and environmental quality improvement should be considered in a scale‐dependent framework. Future research should further examine long‐term associations between cascade dam operation and zooplankton functional diversity, as well as the combined influences of land use change and climate variability on riverine community stability across spatial scales.

## Author Contributions

**Xufei Jiang:** conceptualization (equal), data curation (equal), formal analysis (equal), methodology (equal), writing – original draft (equal). **Rixiu Zhou:** data curation (equal), investigation (equal), methodology (equal), software (supporting). **Wenzhang Dai:** data curation (equal), investigation (equal), validation (equal). **Yipeng Yao:** formal analysis (equal), software (equal), validation (supporting). **Yan Liu:** investigation (equal), resources (equal), validation (equal). **Tianyi Sun:** data curation (supporting), formal analysis (supporting), methodology (supporting). **Jiachen Shen:** data curation (supporting), software (supporting). **Shuqing An:** resources (equal), writing – review and editing (equal). **Lei Yan:** project administration (equal), resources (supporting). **Handan Zhang:** project administration (supporting), resources (equal). **Xin Leng:** funding acquisition (equal), project administration (equal), supervision (equal), writing – review and editing (equal). **Zhi Ding:** conceptualization (supporting), supervision (equal), writing – review and editing (supporting).

## Funding

This work was supported by Chinese Polar Environment Comprehensive Investigation and Assessment Programmes, 2022YFC3204304, and Zhejiang Provincial Natural Science Foundation of China under Grant No. LTGS24D010001 and No. ZCLQN26E0902.

## Conflicts of Interest

The authors declare no conflicts of interest.

## Supporting information


**Figure S1:** One‐way ANOVA for upstream, midstream and downstream of each dam, for abundance and shannon diversity, respectively.
**Figure S2:** Environmental disparities between the dam‐regulated rivers and the control group in the Shaying River Basin. (A) Comparison of water quality between the dam‐regulated sampling points and the control group, revealing significant intergroup differences. (B) Comparison of heavy metal content between the dam‐regulated sampling points and the control group. (C) Kringing interpolation depicting water quality across the entire watershed. (D) Visual representation of the variations in heavy metal content across the entire watershed.
**Table S1:** Tukey HSD test for different locations of basin. Up means upstream of cascade dams, Mid represents midstream and Down means downstream, Approximate probabilities for Post Hoc Tests df = 60. Table shows *p*‐values and red color indicates a significant correlation.
**Table S2:** Zooplankton community composition at the local scale, including abundance, number of species, and biomass of protozoans, rotifers, copepods, and cladocerans. U denotes upstream of the dam, M denotes midstream (reservoir center) of the dam, and D denotes downstream of the dam. UC denotes upstream control reach, MC denotes midstream control reach, and DC denotes downstream control reach of the basin.

## Data Availability

All data underlying the conclusions of this manuscript are deposited in the Zenodo repository (permanent DOI: https://doi.org/10.5281/zenodo.19176480). The dataset is now openly accessible via the following link: https://zenodo.org/records/19176480?token=eyJhbGciOiJIUzUxMiIsImlhdCI6MTc3NDIzNjc0MCwiZXhwIjoxNzgyNzc3NTk5fQ.eyJpZCI6IjgzYWYxNjQxLTQ0NDEtNDg4ZS1hOWZiLTc5OWNkMjIwMmMwZiIsImRhdGEiOnt9LCJyYW5kb20iOiJlZGZiMDIyNGFlNzQ2OTFjOGVkZTUwMGJmNjliODQ1MiJ9.lXZ_dSCwMMCuWo7BDi7Wr9n6ARm3yjzE4Q4mtJp_u5Q7jgE‐3dhpZlXBDXwHLTy7zjNI7e6aeF0tU2A5‐ZZOAw. For data‐related queries, please contact the author at jiangxufei2016@163.com.
