# Supplementary material for: Beyond the Cascade Dams: Scale‐Dependent Effects on Zooplankton Stability in River Ecosystems
Source: Ecol Evol. 2026 Aug 12;16(8):e74019. doi: 10.1002/ece3.74019 (PMC13469334; doi:10.1002/ece3.74019)
Supplement: Supplementary file 1 [file ECE3-16-e74019-s001.docx]

***Supplementary Material***

**Beyond the Cascade Dams: Scale-Dependent Effects on Zooplankton Stability in River Ecosystems**

Xufei Jiang^a b c^, Rixiu Zhou^b^, Wenzhang Dai^b^, Yipeng Yao^b^, Yan Liu^b^, Tianyi Sun^b^, Jiachen Shen^b^, Shuqing An^b^, Lei Yan ^c^, Handan Zhang ^a^, Xin Leng^b*^, Zhi Ding ^b*^

^a^ Institute of Water Sciences, Zhejiang University of Water Resources and Electric Power, Hangzhou, China

^b^ School of Life Science and Institute of Wetland Ecology, Nanjing University, Nanjing, China.

^c^ Qiantang River Basin Center of Zhejiang Province, Hangzhou, China

***Correspondence:**
Xin Leng: [lengx@nju.edu.cn](mailto:lengx@nju.edu.cn) Zhi Ding: dingzhi@nju.edu.cn

Figure S1. one-way ANOVA for upstream, midstream and downstream of each dam, for abundance and shannon diversity, respectively.

Figure S2. Environmental disparities between the dam-regulated rivers and the control group in the Shaying River Basin. (A) Comparison of water quality between the dam-regulated sampling points and the control group, revealing significant intergroup differences. (B) Comparison of heavy metal content between the dam-regulated sampling points and the control group. (C) Kringing interpolation depicting water quality across the entire watershed. (D) Visual representation of the variations in heavy metal content across the entire watershed.

Table S1. Tukey HSD test for different locations of basin. Up means upstream of cascade dams, Mid represents midstream and Down means downstream, Approximate probabilities for Post Hoc Tests df = 60. Table shows p-values and red color indicates a significant correlation.

| **WQ** | **PH** | **DO** | **WT** | **EC** | **ORP** | **TSS** | **NTU** | **RP** | **TP** | **TN** | **NH_4_^+^-N** | **COD** | **Hg** | **Pb** | **Cd** | **As** | **Zn** | **Cu** | **Fe** | **Cr** | **Al** |
| --- | --- | --- | --- | --- | --- | --- | --- | --- | --- | --- | --- | --- | --- | --- | --- | --- | --- | --- | --- | --- | --- |
| Up-Down | 0.990 | 0.854 | 0.933 | 0.000 | 0.752 | 0.000 | 0.001 | 0.009 | 0.002 | 0.016 | 0.031 | 0.412 | 0.787 | 0.440 | 0.680 | 0.000 | 0.942 | 0.999 | 0.083 | 0.000 | 0.100 |
| Up-Mid | 0.972 | 0.962 | 0.978 | 0.000 | 0.257 | 0.000 | 0.063 | 0.712 | 0.119 | 0.000 | 0.149 | 0.916 | 0.595 | 0.950 | 0.035 | 0.000 | 0.755 | 0.719 | 0.187 | 0.001 | 0.161 |
| Mid-Down | 0.925 | 0.671 | 0.826 | 0.001 | 0.053 | 0.036 | 0.102 | 0.033 | 0.158 | 0.285 | 0.611 | 0.578 | 0.966 | 0.554 | 0.240 | 0.129 | 0.933 | 0.752 | 0.814 | 0.393 | 0.901 |

Table S2. Zooplankton community composition at the local scale, including abundance, number of species, and biomass of protozoans, rotifers, copepods, and cladocerans. U denotes upstream of the dam, M denotes midstream (reservoir center) of the dam, and D denotes downstream of the dam. UC denotes upstream control reach, MC denotes midstream control reach, and DC denotes downstream control reach of the basin.

|  | Abundance | | | | | Number of species | | | | | Biomass | | | | |
| --- | --- | --- | --- | --- | --- | --- | --- | --- | --- | --- | --- | --- | --- | --- | --- |
| Plot | Protozoans | Rotifers | Copepods | Cladocerans | Total | Protozoans | Rotifers | Copepods | Cladocerans | Total | Protozoans | Rotifers | Copepods | Cladocerans | Total |
| ZPT_U | 404.16 | 37.09 | 7.21 | 3.47 | 451.93 | 13 | 27 | 9 | 1 | 50 | 0.01 | 0.01 | 0.20 | 0.43 | 0.64 |
| ZPT_M | 1555.40 | 48.29 | 2.57 | 9.62 | 1615.88 | 18 | 29 | 5 | 4 | 56 | 0.05 | 0.01 | 0.08 | 0.97 | 1.11 |
| ZPT_D | 4706.48 | 718.12 | 6.86 | 46.32 | 5477.78 | 26 | 53 | 5 | 3 | 87 | 0.35 | 0.38 | 0.19 | 5.09 | 6.01 |
| BGS_U | 3447.92 | 883.49 | 13.93 | 45.00 | 4390.34 | 17 | 52 | 7 | 2 | 78 | 0.12 | 0.23 | 0.30 | 4.81 | 5.45 |
| BGS_M | 3403.30 | 844.60 | 34.74 | 23.92 | 4306.56 | 20 | 43 | 5 | 4 | 72 | 0.40 | 0.25 | 1.13 | 3.62 | 5.40 |
| BGS_D | 4807.42 | 878.04 | 15.18 | 11.16 | 5711.80 | 14 | 38 | 6 | 2 | 60 | 0.19 | 0.64 | 0.46 | 1.19 | 2.47 |
| UC | 231.84 | 53.71 | 0.00 | 1.14 | 286.69 | 10 | 30 | 3 | 1 | 44 | 0.08 | 0.03 | 0.00 | 0.14 | 0.25 |
| LH_U | 5558.48 | 504.03 | 117.45 | 74.29 | 6254.25 | 13 | 35 | 8 | 6 | 62 | 0.20 | 0.36 | 3.30 | 11.82 | 15.68 |
| LH_M | 1611.00 | 347.50 | 77.80 | 67.87 | 2104.17 | 15 | 27 | 7 | 9 | 58 | 0.16 | 0.17 | 2.33 | 9.41 | 12.07 |
| LH_D | 5161.60 | 306.88 | 0.00 | 19.17 | 5487.64 | 18 | 39 | 4 | 4 | 65 | 0.23 | 0.27 | 0.00 | 1.66 | 2.17 |
| MC | 2256.80 | 123.27 | 2.25 | 0.00 | 2382.32 | 23 | 41 | 4 | 0 | 68 | 0.19 | 0.09 | 0.07 | 0.29 | 0.64 |
| ZK_U | 8213.20 | 1007.95 | 19.78 | 24.73 | 9265.65 | 22 | 49 | 6 | 3 | 80 | 0.34 | 1.55 | 0.55 | 3.11 | 5.56 |
| ZK_M | 7821.56 | 1885.40 | 31.17 | 1.96 | 9740.09 | 22 | 57 | 7 | 1 | 87 | 0.44 | 3.22 | 0.82 | 1.38 | 5.87 |
| ZK_D | 10732.48 | 1675.00 | 11.14 | 0.00 | 12418.62 | 17 | 52 | 5 | 0 | 74 | 0.71 | 1.64 | 0.30 | 0.17 | 2.82 |
| XC_U | 7417.00 | 4653.60 | 8.80 | 1.00 | 12080.40 | 15 | 51 | 6 | 1 | 73 | 0.25 | 3.05 | 0.14 | 1.20 | 4.63 |
| XC_M | 2919.80 | 3357.92 | 9.20 | 9.20 | 6296.12 | 19 | 59 | 4 | 2 | 84 | 0.07 | 2.33 | 0.25 | 2.63 | 5.29 |
| XC_D | 5070.80 | 1037.48 | 5.22 | 0.00 | 6113.50 | 23 | 48 | 3 | 0 | 74 | 0.34 | 1.18 | 0.15 | 0.63 | 2.30 |
| YQ_U | 2024.32 | 1091.44 | 82.40 | 48.62 | 3246.78 | 14 | 47 | 11 | 3 | 75 | 0.04 | 4.30 | 2.18 | 8.38 | 14.90 |
| YQ_M | 2442.00 | 3497.20 | 98.10 | 112.70 | 6150.00 | 14 | 66 | 12 | 5 | 97 | 0.07 | 3.35 | 2.38 | 11.75 | 17.55 |
| YQ_D | 954.00 | 1306.30 | 57.40 | 26.30 | 2344.00 | 14 | 56 | 3 | 4 | 77 | 0.12 | 1.13 | 1.63 | 2.44 | 5.32 |
| DC | 3200.00 | 1612.70 | 246.20 | 128.90 | 5187.80 | 16 | 44 | 8 | 5 | 73 | 0.41 | 1.67 | 5.71 | 7.28 | 15.07 |
| FY_U | 4512.77 | 538.46 | 47.55 | 11.24 | 5110.02 | 16 | 46 | 8 | 4 | 74 | 0.17 | 1.71 | 1.71 | 3.75 | 7.34 |
| FY_M | 3606.00 | 2880.80 | 348.40 | 9.20 | 6844.40 | 16 | 55 | 12 | 3 | 86 | 0.15 | 9.44 | 8.58 | 17.04 | 35.21 |
| FY_D | 18544.00 | 6154.95 | 7.30 | 6.00 | 24712.25 | 19 | 49 | 2 | 1 | 71 | 0.56 | 2.91 | 0.10 | 2.01 | 5.59 |
